# Large language models in radiology exams: A cross-sectional comparative analysis of performance in Turkish and English

**DOI:** 10.1097/MD.0000000000049517

**Published:** 2026-06-26

**Authors:** Şahinde Atlanoğlu, Mehmet Ali Gedik

**Affiliations:** a Department of Radiology, Kütahya Health Sciences University, Kütahya, Türkiye.

**Keywords:** artificial intelligence, ChatGPT, Claude Sonnet, Google Gemini, Grok, large language models, radiology

## Abstract

This study evaluated the success of large language models on radiology questions, analyzing language variations, temporal consistency, and performance against residents. We evaluated ChatGPT-5, Grok-4, Claude 4.5 Sonnet, and Gemini 2.5 Pro using 100 multiple-choice questions across 5 subspecialties. Linguistic impact (Turkish vs English) and 1-week temporal reliability were assessed. Performance was benchmarked against a control group of 18 radiology residents (years 1–3). Gemini 2.5 Pro achieved the highest accuracy (90%), followed by Claude 4.5 Sonnet (86%). All models and 3rd-year residents significantly outperformed junior residents. While no significant performance gap existed between languages (*P* = 1.000), Claude 4.5 Sonnet demonstrated superior temporal reliability (κ = 0.872) compared with the moderate consistency of Grok-4 and ChatGPT-5. High-performance large language models provide accurate radiology knowledge comparable with senior residents, showing significant potential for education. Future research must incorporate image-based datasets to determine clinical efficacy.

## 1. Introduction

The incorporation of artificial intelligence (AI) into clinical practice has become a focal point in recent medical scholarship.^[1]^ Within this domain, ChatGPT has emerged as a prominent large language model (LLM), notable for its swift uptake and transformative influence.^[2]^ LLMs employ natural language processing architectures that generate contextual and dynamic responses to complex textual queries by leveraging extensive datasets, deep learning algorithms, and transformer frameworks. Unlike rule-based systems, these models demonstrate sophisticated reasoning capabilities through the identification and interpretation of semantic relationships in input data.^[3]^ Their applications span academic research, clinical decision support, and medical education.^[4]^

Although AI in diagnostic radiology has historically centered on image analysis, recent progress with LLMs has broadened their use in text-heavy tasks, such as differential diagnosis, disease categorization, and radiological instruction.^[5]^ Given the critical importance of precise diagnoses in radiology and escalating demand for automation, integrating these technologies into routine workflows is essential. Scientific endorsement of such integration depends on LLMs achieving diagnostic accuracy that matches or surpasses that of radiologists and residents.^[6]^ Despite the explosion of LLM research, few studies have assessed radiology-specific expertise or benchmarked model performance against human proficiency. This investigation aimed to deliver a thorough evaluation of the theoretical capabilities, linguistic variations, and temporal stability of contemporary LLMs in the field of radiology.

## 2. Methods

### 2.1. Question set creation and content validity

Ethics committee approval was not sought for this study because it did not involve human participants, patient data, or any identifiable personal information. The study evaluated only the responses generated by LLMs and the anonymized performance of volunteer radiology residents who completed a question set as a non-clinical benchmark; under applicable national regulations, such research does not require institutional review board or ethics committee approval or informed consent.

A set of 100 multiple-choice items was assembled for this investigation, sourced from a widely used core radiology textbook^[7]^ and a standard board exam question bank.^[8]^ Two senior radiologists, each with more than 10 years of clinical experience, crafted questions to provide an even distribution across 5 subspecialties: thorax, neuroradiology, musculoskeletal system, abdomen, and interventional radiology, with 20 items per domain. The non-visual questions were subsequently translated into English by a certified translation agency and subjected to linguistic validation to guarantee precision and consistency.

### 2.2. LLMs and application protocol

This study employed paid subscriptions of ChatGPT-5, Grok-4, Claude 4.5 Sonnet, and Gemini 2.5 Pro as the primary analytical instruments. Each question was submitted individually to the respective model interfaces in distinct sessions to reduce potential response bias. For an initial performance comparison, questions were presented to radiology residents in Turkish, whereas the language models received them in English.

### 2.3. Comparative analysis design

The study was structured across 3 primary comparative layers:

### 2.4. Language-based performance

The questions were administered to the ChatGPT and Gemini models in both Turkish and English to assess the influence of language on model success rates.

### 2.5. Temporal reliability

The full question set was resubmitted after a 1-week interval to the ChatGPT, Grok, and Claude models to assess response stability over time.

### 2.6. Version comparison

Questions were posed in English to successive releases of each model (ChatGPT v5.1, v5.0, v4.0, Gemini 2.5 Pro, and 2.5 Flash) to identify performance shifts attributable to model updates.

### 2.7. Statistical analysis

The data were analyzed using IBM SPSS version 23. To compare the accuracy rates between the 2 different AI models, Yates correction was applied. For comparisons across multiple AI models and residents, the Pearson chi-square test and Fisher exact test with Monte Carlo correction were used. Pairwise comparisons were conducted using the *Z*-test with Bonferroni correction. The McNemar test assessed differences in correct response rates between the 2 versions of the same AI model, between responses in the 2 languages, and between repeated measurements. The Cochran *Q* test was employed to examine the differences in accuracy among the 3 versions of the same model.

Agreement between AI models, responses in different languages, and repeated responses was evaluated using the Kappa test. The Fleiss Kappa test was used to assess the agreement among responses from the 3 different AI models. Categorical variables are reported as frequency (percentage). Statistical significance was set at *P* < .050. Kappa agreement levels were interpreted according to the Landis and Koch (1977) classification.

## 3. Results

Among the 100 radiology questions analyzed, Gemini 2.5 Pro achieved the highest success rate among the LLMs, with 90 correct responses. When radiology residents were grouped by year of residence, the correct response rates were 75.8% for 3rd-year residents, 66% for 2nd-year residents, and 58.7% for 1st-year residents. Subspecialty analysis revealed that 3rd-year residents significantly outperformed 1st-year residents in musculoskeletal radiology. In abdominal radiology, Claude 4.5 Sonnet and Gemini 2.5 Pro demonstrated superior performance compared with first-year residents. Overall, the success rates of all LLMs and 3rd-year residents were statistically higher than those of the 1st- and 2nd-year residents across the entire question set (Table 1).

**Table 1:** Performance comparison of large language models and radiology residents: total and subspecialty accuracy rates.

		LLM							Test statistic	*P*
		Grok 4	Claude Sonnet 4.5	ChatGPT5	Gemini 2.5 Pro	3rd year	2nd year	1st year		
Interventional	False	3 (15)	4 (20)	4 (20)	3 (15)	45 (28.1)	27 (45)	58 (41.4)	18,483	**.005** *
	True	17 (85)	16 (80)	16 (80)	17 (85)	115 (71.9)	33 (55)	82 (58.6)		
Musculoskeletal	False	6 (30)	2 (10)	4 (20)	2 (10)	36 (22.5)	19 (31.7)	57 (40.7)	20,253	**.002** *
	True	14 (70)†‡	18 (90)†‡	16 (80)†‡	18 (90)†‡	124 (77.5)‡	41 (68.3)†‡	83 (59.3)†		
Neuro	False	4 (20)	6 (30)	3 (15)	3 (15)	47 (29.4)	19 (31.7)	62 (44.3)	15,958	**.011** *
	True	16 (80)	14 (70)	17 (85)	17 (85)	113 (70.6)	41 (68.3)	78 (55.7)		
Thorax	False	1 (5)	1 (5)	1 (5)	2 (10)	34 (21.3)	18 (30)	52 (37.1)	28,275	**<.001** *
	True	19 (95)	19 (95)	19 (95)	18 (90)	126 (78.8)	42 (70)	88 (62.9)		
Abdomen	False	2 (10)	1 (5)	3 (15)	1 (5)	32 (20)	19 (31.7)	60 (42.9)	36,867	**<.001** *
	True	18 (90)†‡	19 (95)‡	17 (85)†‡	19 (95)‡	128 (80)‡	41 (68.3)†‡	80 (57.1)†		
Total	False	**16 (16**)	**14 (14**)	**15 (15**)	**11 (11**)	**194 (24.3**)	**102 (34**)	**289 (41.3**)	107,795	**<.001** §
	True	**84 (84**)†	**86 (86**)†	**85 (85**)†	**89 (89**)†	**606(75.8**)†	**198 (66**)‡	**411 (58.7**)‡		

Frequency (percent).

* Monte Carlo Corrected Fisher Exact.

† ,‡ There is no significant difference between groups sharing the same symbol.

§ Pearson chi-square test.

To assess the reliability and temporal consistency of the LLMs, the same set of questions was administered to Grok-4, Claude 4.5 Sonnet, and ChatGPT-5 in 2 separate sessions, 1 week apart. The agreement between model responses in the initial and follow-up sessions was evaluated using Cohen kappa coefficient. The analysis demonstrated that Claude 4.5 Sonnet achieved a “very good” level of internal consistency (kappa = 0.872). In contrast, Grok-4 and ChatGPT-5 exhibited “moderate” agreement (kappa = 0.575 and kappa = 0.559, respectively; Table 2).

**Table 2:** Temporal accuracy comparison and agreement analysis of large language models.

	Grok 4	Claude Sonnet 4.5	ChatGPT5
1st assessment			
False	16 (16)	14 (14)	15 (15)
True	84 (84)	86 (86)	85 (85)
2nd assessment			
False	18 (18)	13 (13)	11 (11)
True	82 (82)	87 (87)	89 (89)
McNemar *P*	.774	1.000	.344
Kappa/*P*	0.575/**<**.001	0.872/**<**.001	0.559/**<**.001

Frequency (percentage).

**Figure 1.**
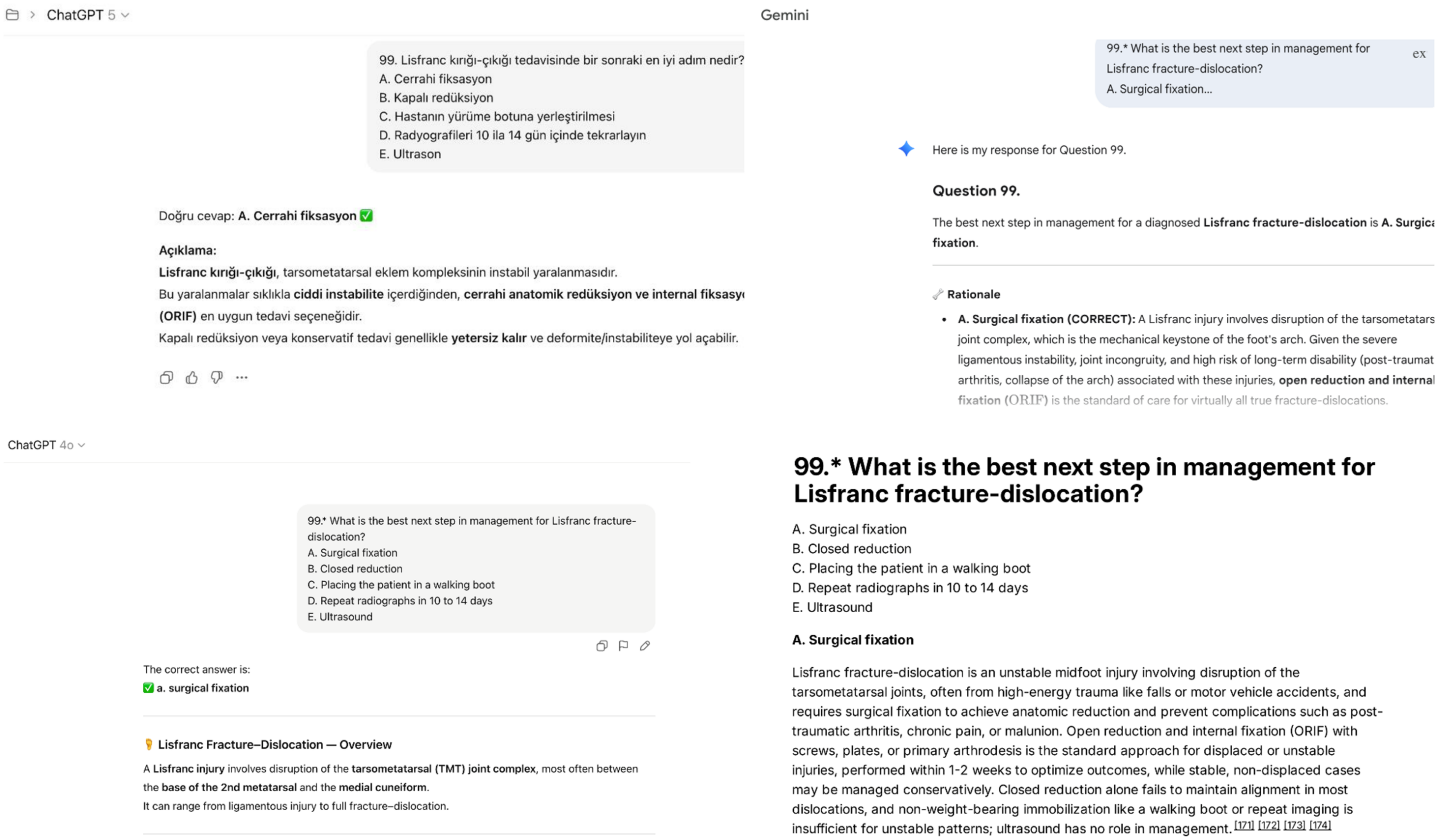
Answers to the same question from ChatGPT-5 (Turkish) and ChatGPT-4-Gemini-Grok LLMs (English). All answered correctly.

**Figure 2.**
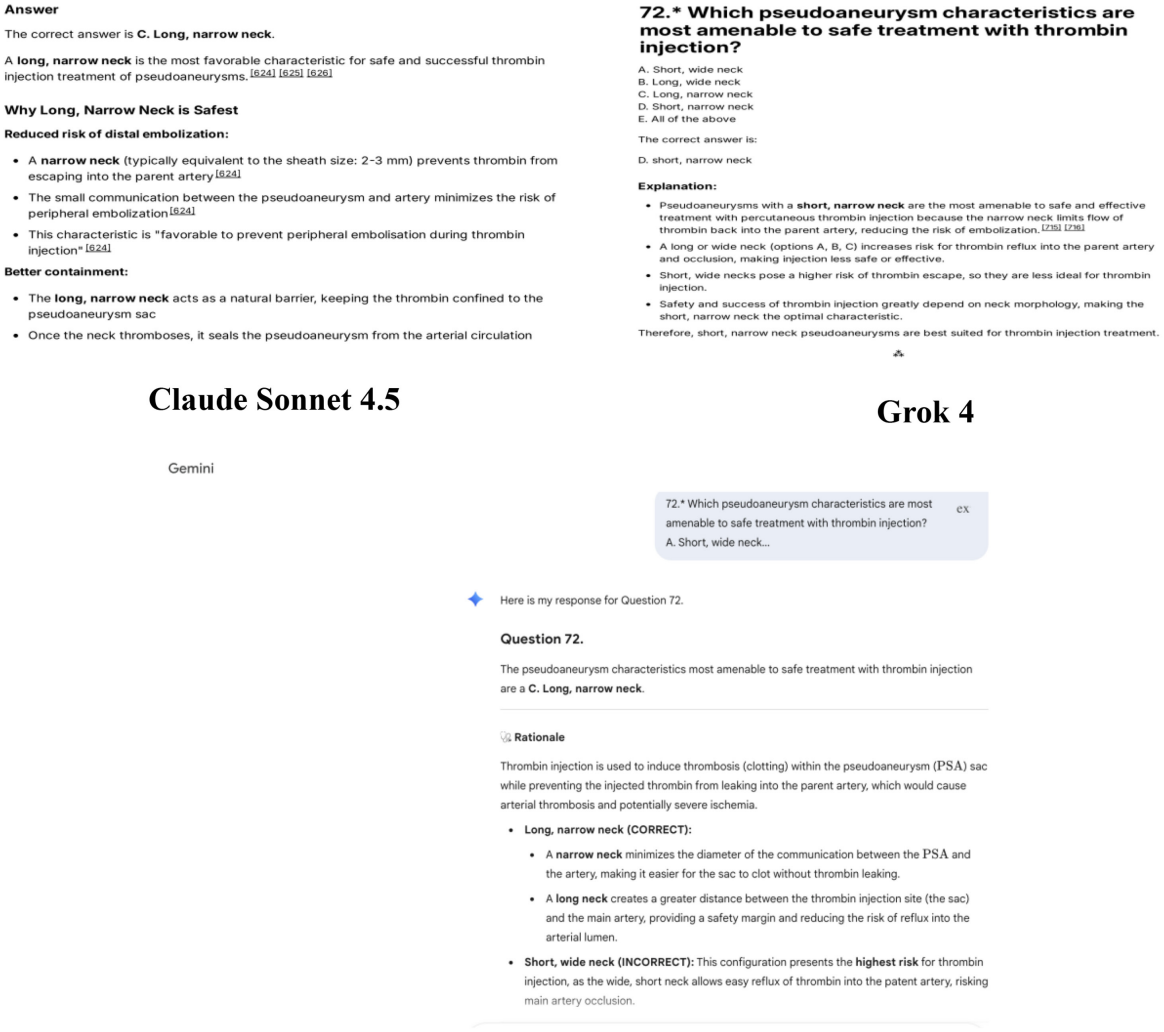
Answers to the same question from Gemini and Claude Sonnet, Grok 4: Grok 4’s answer was incorrect.

To evaluate language-based performance variations, questions were administered separately in Turkish and English to ChatGPT-5 and Gemini 2.5 Pro (Figs. 1 and 2). The analysis revealed no statistically significant difference in success rates between the 2 languages in either model (McNemar, *P* = 1.000). Assessment of the agreement between responses in different languages showed that both ChatGPT-5 (kappa = 0.733) and Gemini 2.5 Pro (kappa = 0.734) demonstrated a “good” level of linguistic consistency (Table 3).

**Table 3:** Evaluation of accuracy and agreement in large language models’ responses to English and Turkish questions.

	LLM		Test statistics (χ^2^)	*P* *
	ChatGPT5	Gemini2.5		
English				
False	16 (16)	10 (10)	1.105	.293
True	84 (84)	90 (90)		
Turkish				
False	15 (15)	11 (11)	0.398	.528
True	85 (85)	89 (89)		
McNemar	1.000	1.000		
Kappa/*P*	0.733/**<**.001	0.734/**<**.001		

Frequency (percentage).

* Yates correction.

In the comparative analysis across different versions of ChatGPT, no statistically significant difference was observed in the overall accuracy rates (*P* = .282). Fleiss Kappa analysis, conducted to assess response consistency among the versions, revealed a “moderate” level of agreement (kappa = 0.600; *P* < .001; Table 4).

**Table 4:** Comparison of accuracy and evaluation of agreement for responses to questions asked across ChatGPT versions.

	ChatGPT			Fleiss Kappa/*P*	Cochran *Q*	*P*
	ChatGPT5	ChatGPT4	ChatGPT5.1			
False	15 (15)	17 (17)	12 (12)	0.600/**<**.001	2.533	.282
True	85 (85)	83 (83)	88 (88)			

Frequency (percentage).

In the comparison between different versions of the Gemini model, the rates of correct responses were statistically similar, with no significant performance difference observed (*P* = .508). Response-based consistency analysis revealed a “moderate” level of agreement between versions (kappa = 0.589; *P* < .001; Table 5).

**Table 5:** Comparison of accuracy and evaluation of agreement for responses to questions asked across Gemini versions.

	Gemini		Kappa/*P*	McNemar
	Gemini 2.5 Pro	Gemini 2.5 Flash		
False	11 (11)	14 (14)	0.589/**<**.001	0.508
True	89 (89)	86 (86)		

Frequency (percentage).

## 4. Discussion

LLMs transform medical decision support processes by leveraging deep learning architectures to analyze extensive natural language data.^[4]^ The rapid evolution of AI algorithms has steadily increased their potential for integration into clinical applications.^[9]^ Unlike previous studies, this research stands out by simultaneously evaluating 4 contemporary models and conducting a cross-linguistic analysis to compare the Turkish and English performance of the 2 leading models.

The success rates observed in our study, ranging from 84% to 90%, represent a notable improvement over earlier findings. Previous studies have reported lower accuracy rates: 40.8% to 62.6% for ChatGPT-3.5, 65% to 81% for ChatGPT-4, 64.89% to 88.1% for ChatGPT-4o, 38.8% for Google Bard, 55.73% for Google Gemini, and 85.7% for Gemini Advanced.^[2,3,10–12]^ The 85% success rate achieved by ChatGPT-5 in our study indicates a significant enhancement in clinical reasoning compared with earlier versions, GPT-3.5, and GPT-4.^[13]^ Similarly, the 90% success rate of Gemini 2.5 Pro highlights substantial technological progress, likely owing to its access to up-to-date datasets.^[4]^ Nevertheless, as radiology practice fundamentally relies on visual data analysis, current text-based models alone are insufficient for direct clinical use. The future role of AI in radiology will be better defined using models with image interpretation capabilities. Promising developments in medicine-specific structured multimodal LLMs, such as Google’s Med-PaLM M and Microsoft’s LLaVA-Med, show highly encouraging results for radiological diagnostics.^[14,15]^

In this study, Gemini 2.5 Pro and Claude Sonnet achieved the highest success rates in abdominal and musculoskeletal radiology, whereas Gemini 2.5 Pro and Grok-4 performed best in interventional radiology. In neuroradiology, Gemini 2.5 Pro and ChatGPT-5 were the top performers, while Claude Sonnet, Grok-4, and ChatGPT-5 excelled in thoracic radiology. The 85% success rates for both ChatGPT-5 and Gemini 2.5 Pro in neuroradiology were notably higher than those reported by Gupta et al.^[10]^ Our musculoskeletal and abdominal radiology results also significantly surpassed the findings of Huang et al.^[11]^ Payne et al^[13]^ noted that ChatGPT-4 performed between 1st- and 2nd-year residents (61.9%); in contrast, our study found that Claude Sonnet and Gemini Pro outperformed 1st-year residents in abdominal radiology, and overall, LLMs and 3rd-year residents achieved higher success rates than 1st- and 2nd-year residents. These results differ from those of Sarangi et al., who reported that 2 senior residents (63.33% and 57.5%) outperformed several AI models (Bard and ChatGPT-3.5).^[12]^ The strong performance of LLMs on non-visual questions highlights their potential for integration into radiology. However, for these models to serve as reliable assistants in image interpretation and complex diagnostic processes – the core of radiology practice – further evolution of LLM architecture is necessary. Specifically, the development of multimodal capabilities is essential for effective deployment in clinical decision support systems.^[4,16]^

In this study, all questions were presented to ChatGPT-5 and Gemini 2.5 Pro in both Turkish and English to evaluate the impact of language on model performance. ChatGPT-5 achieved 84 correct responses for English and 85 for Turkish, while Gemini 2.5 Pro scored 90 for English and 89 for Turkish. These findings contrast with those of Toyama et al,^[4]^ who attributed lower performance in Japanese to structural complexity. In our study, the similar success rates in both languages, despite the models’ primary training on English datasets, reflect advancements in multilingual processing and language recognition in next-generation models. Meddeb et al^[17]^ found that GPT-4 excelled in translating radiology reports from English to German, Greek, Thai, and Turkish, while GPT-3.5 performed best for French. Although these models demonstrated high clarity and semantic consistency, their medical terminology accuracy remained moderate, highlighting the need for further research across diverse language families and technical fields.

Is et al^[18]^ observed that ChatGPT-4o and Google Gemini changed responses when questions were repeated, whereas Brin et al^[9]^ noted that ChatGPT-3.5 altered answers for 82.5% of repeated questions, but ChatGPT-4 did not. In our study, Grok-4 and ChatGPT-5 showed moderate agreement between the initial and follow-up responses (kappa = 0.575 and kappa = 0.559), whereas Claude Sonnet exhibited very good agreement (kappa = 0.872). Literature frequently highlights the risk of erroneous or fabricated outputs, known as “hallucinations,” in LLMs.^[2,19]^ The moderate to very good agreement coefficients in our study suggest a decreasing tendency for hallucinations and an increasing response stability in next-generation models.

Integrating ChatGPT into healthcare applications entails significant risks and limitations beyond its benefits. Ensuring system reliability requires careful management of ethics-based risks, including data security, patient privacy, and algorithmic bias.^[3]^ The limitations of our study include the exclusive use of non-visual, multiple-choice questions and a restricted question pool of 100, derived from only 2 textbooks.

Large-language models demonstrated high-accuracy performance on fundamental radiology questions, indicating their potential as supportive tools in medical education for providing feedback and optimizing learning processes. Future studies incorporating radiological images are essential to clarify the role of LLMs in clinical radiology. For full integration into radiology practice, the success of these models in image evaluation must match their strong performance in theoretical examination.

## Author contributions

**Conceptualization:** Şahinde Atlanoğlu, Mehmet Ali Gedik.

**Data curation:** Şahinde Atlanoğlu.

**Formal analysis:** Şahinde Atlanoğlu.

**Investigation:** Şahinde Atlanoğlu, Mehmet Ali Gedik.

**Methodology:** Şahinde Atlanoğlu, Mehmet Ali Gedik.

**Project administration:** Şahinde Atlanoğlu.

**Software:** Şahinde Atlanoğlu.

**Validation:** Şahinde Atlanoğlu, Mehmet Ali Gedik.

**Resources:** Mehmet Ali Gedik.

**Supervision:** Mehmet Ali Gedik.

**Writing – review & editing:** Mehmet Ali Gedik.

**Writing – original draft:** Şahinde Atlanoğlu.
